# Geophysical precursors of the July-August 2019 paroxysmal eruptive phase and their implications for Stromboli volcano (Italy) monitoring

**DOI:** 10.1038/s41598-020-67220-1

**Published:** 2020-06-24

**Authors:** Flora Giudicepietro, Carmen López, Giovanni Macedonio, Salvatore Alparone, Francesca Bianco, Sonia Calvari, Walter De Cesare, Dario Delle Donne, Bellina Di Lieto, Antonietta M. Esposito, Massimo Orazi, Rosario Peluso, Eugenio Privitera, Pierdomenico Romano, Giovanni Scarpato, Anna Tramelli

**Affiliations:** 1 Istituto Nazionale di Geofisica e Vulcanologia, Osservatorio Vesuviano, Napoli, Italy; 2 Observatorio Geofísico Central, Instituto Geográfico Nacional (IGN), Madrid, Spain; 3 Istituto Nazionale di Geofisica e Vulcanologia, Osservatorio Etneo, Catania, Italy

**Keywords:** Natural hazards, Solid Earth sciences

## Abstract

Two paroxysmal explosions occurred at Stromboli volcano in the Summer 2019, the first of which, on July 3, caused one fatality and some injuries. Within the 56 days between the two paroxysmal explosions, effusive activity from vents located in the summit area of the volcano occurred. No significant changes in routinely monitored parameters were detected before the paroxysmal explosions. However, we have calculated the polarization and the fractal dimension time series of the seismic signals from November 15, 2018 to September 15, 2019 and we have recognized variations that preceded the paroxysmal activity. In addition, we have defined a new parameter, based on RSAM estimation, related to the Very Long Period events, called *VLP size*, by means of which we have noticed significant variations through the whole month preceding the paroxysm of July 3. In the short term, we have analyzed the signals of a borehole strainmeter installed on the island, obtaining automatic triggers 10 minutes and 7.5 minutes before the July 3 and the August 28 paroxysms, respectively. The results of this study highlight mid-term seismic precursors of paroxysmal activity and provide valuable evidence for the development of an early warning system for paroxysmal explosions based on strainmeter measurements.

## Introduction

**Figure 1:**
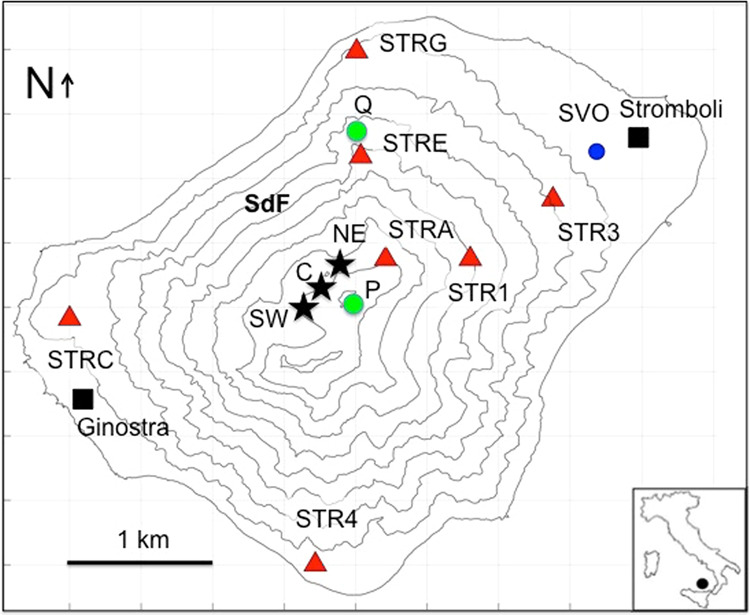
Map of Stromboli Island. The red triangles indicate the stations of the seismic network and the blue circle indicates the SVO strainmeter location. The “Sciara del Fuoco” (SdF) flank, where the ejecta from the persistent explosive activity accumulate, is shown. The black stars indicate the position of the main vent regions: northeast (NE), central (C) and southwest (SW). The location of Stromboli is reported in the inset. The position of the SPT (P), SQT and SQV (Q) monitoring cameras is shown with green circles. The software used to create the map is Matlab R2017a (https://it.mathworks.com/products/new_products/release2017a.html).

Stromboli (Aeolian Archipelago, Italy) is an open conduit volcano with persistent explosive activity. It is located in the Mediterranean Sea, not far from the coasts of Sicily and Calabria (Fig. 1). The persistent explosive Strombolian activity consists of several hundred of moderate-intensity events per day. Typical Strombolian explosions eject pyroclastic fragments at the height of some tens of meters, which fall a short distance from the eruptive vent. Explosions occur in numerous eruptive vents located in the summit area of the volcano that can change over time both in number and position. However, the eruptive vents can be grouped into three areas (Fig. 1), northeast (NE), central (C) and southwest (SW), and are distributed along the dominant structural direction (NE-SW) of a graben-like collapsed area at the top of the volcanic edifice^1–3^.

Major explosions^4,5^ eject pyroclastic material over a hundred meters high, which can fall outside the crater terrace in the area visited by tourists. The frequency of these phenomena varies in time, with an average of 2 events per year^5–7^. Paroxysms, violent explosions that produce eruptive columns more than 3 km high and are often accompanied by pyroclastic flows, can also occur at Stromboli^8–13^. Ballistic blocks associated with these explosions can reach up to 2 m in diameter. Strombolian paroxysms are rare and their occurrence frequency varies over time. Authors in^7^ report 18 paroxysms over 110 years, from historical records.

Effusive phases can also occur in Stromboli, when the magma mass flow rate increases^14,15^. The effusive eruption that marked a turning point in the perception of the risk associated with the eruptive activity of Stromboli was that of 2002-2003, which was characterized by landslides on the “Sciara del Fuoco” slope (SdF, in Fig. 1; December 30, 2002), which is a collapse structure in the northwest flank of the volcano^16^ (Fig. 1). The landslides caused a potentially destructive tsunami along the coasts of the island^17–19^. This effusive eruption was characterized by a paroxysmal explosion that occurred on April 5, 2003^8,11^, the first large-scale paroxysmal eruption after the paroxysm of 1950^20^. Flank eruptions are supposed to be a possible trigger for paroxysmal explosions by decompression of the plumbing system caused by lowering of the magma level within the conduit^21–23^. Another effusive flank eruption occurred in 2007^24–26^ and was also characterized by a paroxysmal explosion on March 15, 2007^10,13^. The last effusive eruption occurred between August and November 2014; it was not accompanied by paroxysmal explosions^27–29^. After this eruption, Stromboli showed a period of low activity until May, 2017 when a reawakening phase began^30^. This phase was characterized by a general increase in the eruptive activity, in terms of number of explosions per hour, seismic signal amplitude and occurrence of modest lava overflows from the eruptive vents, and by the resumption of the major explosions. From July, 2017 to August, 2018 nine major explosions were recorded^30^. During this period, on December 7, 2017, the alert level was raised from green (base) to yellow (attention) and returned to the green level after about three months. Subsequently, a new phase of increase in eruptive activity occurred between November 2018 and January 2019. In this period, precisely on December 24, 2018, the alert level was raised again from green to yellow, but no significant eruptive events such as lava flows or paroxysmal explosions occurred. It returned therefore to the base level on April 4, 2019.

**Figure 2:**
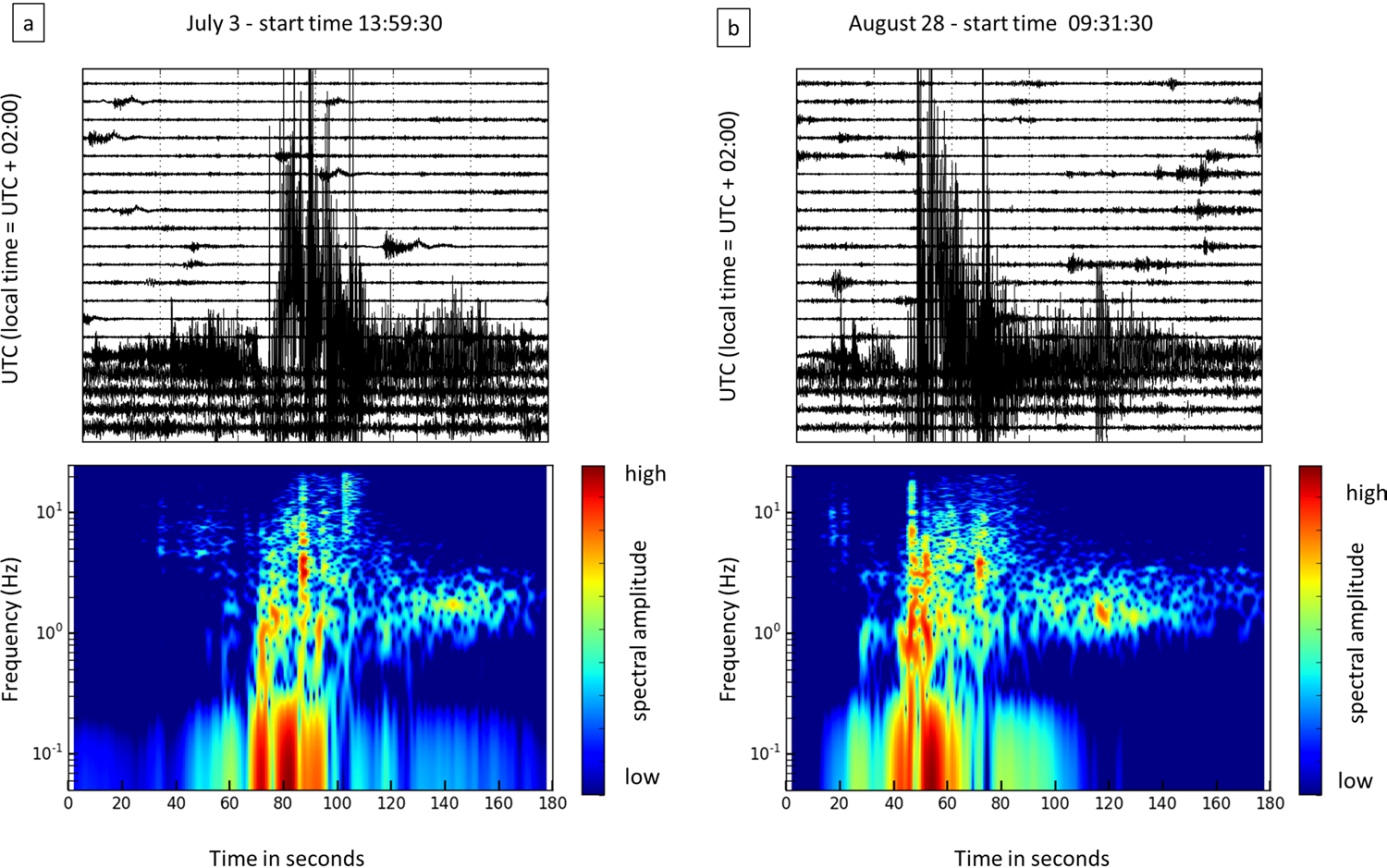
Seismograms and spectrograms of the July 3 (**a**) and of the August 28 (**b**) paroxysms recorded at the STRA station, E-W horizontal component. The top plots represent one hour of seismogram containing the signals of the paroxysmal explosions. Each row shows 3 minutes of signal. The spectrogram is relative to the 3-minute row that contains the paroxysmal explosion signal.

Finally, on July 3, 2019, during a period of apparently moderate activity, a paroxysmal explosion occurred, causing a victim and some injuries. This paroxysm (Fig. 2a) gave rise to an eruptive column more than 5 km high and to a pyroclastic flow that expanded along the SdF slope and traveled about 1 km on the sea surface. This paroxysmal explosion changed the morphology of the summit area and marked the beginning of an effusive phase that lasted about 2 months^31^. On August 28, a second paroxysmal explosion (Fig. 2b) occurred, similar to that of July 3, with another pyroclastic flow that expanded along the same path as the previous one and again traveled about 1 km on the sea surface.

Our analysis focuses on the period of activity from November, 2018 to September, 2019, which of course includes both the eruptive phase reported in July-August, 2019, that was not characterized by changes in monitored parameters before the first paroxysm, and the period between November, 2018 and January, 2019, that was, by contrast, characterized by changes in monitored parameters but no anomalous eruptive activity. These two phases will be here compared. In particular we analyzed the seismic and strainmeter data focusing on parameters that are not routinely monitored and looking for possible precursors of the paroxysms of July 3 and August 28, 2019. These parameters could be useful to mitigate the impact of future violent explosive eruptions on the island. The data we have used in our work come from the seismic network^32^, from the monitoring web-cameras comprising both visible and thermal images^26,29^ and from a Sacks-Evertson borehole strainmeter^13,33^. Since the Strombolian seismic signals, such as VLPs and volcanic tremor, are typically near horizontally polarized^24,34–36^ for the analysis of the seismic amplitude that uses a single seismic channel, e.g. “VLP size”, VLP peak-to-peak amplitude, and Fractal Dimension analysis, we chose the E – W component of the STRA station, being the closest one to the eruptive vents. Details on the instrumentations, the “VLP size” definition and data analysis are shown in the Methods section and the time series of the analyzed data are available as Supplementary Data.

## Results

### Typical volcanic seismicity of Stromboli

**Figure 3:**
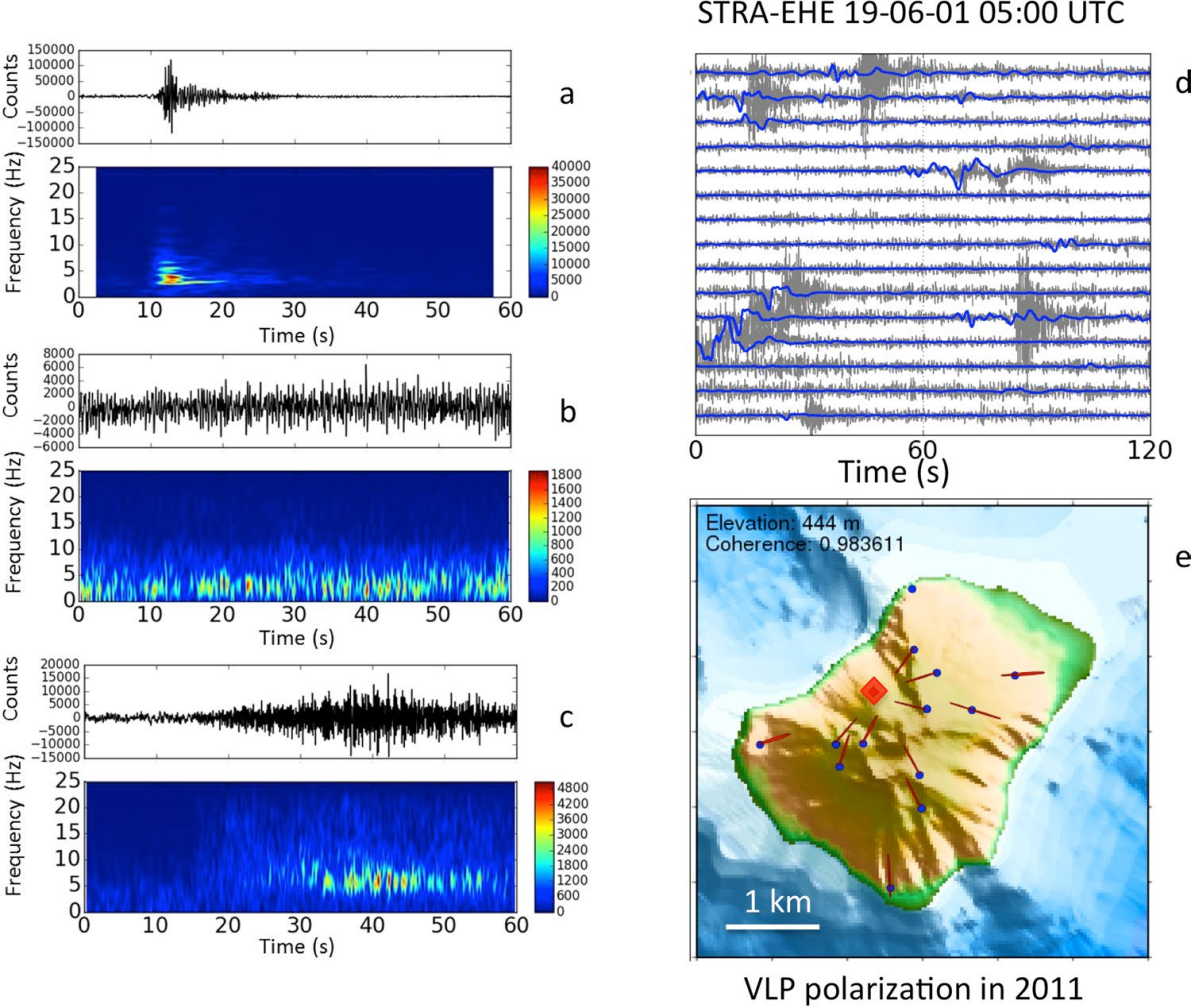
Seismograms and spectrograms of an explosion-quake (**a**), volcanic tremor (**b**), and a landslide signal (**c**) recorded at the STRA station, vertical component. The plot (**d**) shows the VLP events (blue) associated to the explosion-quakes (gray), recorded at the STRA station, E-W horizontal component. The plot (**e**) represents the azimuth mode of the polarization of more than 120,000 VLP events recorded between January 1 and December 31, 2011, when the seismic network had a greater number of stations. The red diamond indicates the VLP source location below SdF. The software used to create the map is GMT 4 (https://www.soest.hawaii.edu/gmt/).

The seismic signals due to the Stromboli volcanic activity are composed of explosion-quakes, caused by explosions^35–38^, volcanic tremor^34,39^, and signals produced by landslides^40,41^, which mobilize the pyroclastic material deposited by the explosive activity on the SdF flank. The explosion-quakes (Fig. 3a) have a frequency content typically below 10 Hz. The Strombolian volcanic tremor shows a frequency content mainly concentrated in 1-3 Hz band (Fig. 3b), whereas the signals due to landslides have a higher frequency range (Fig. 3c). The explosion-quakes contain Very Long Period (VLP) events (0.05-0.5 Hz frequency band) (Fig. 3d) that are typically polarized in a radial direction with respect to the source^35^ (Fig. 3e). Moreover, signals generated by the puffing and spattering activity at the active vents contribute to the seismic wave field^39^.

**Figure 4:**
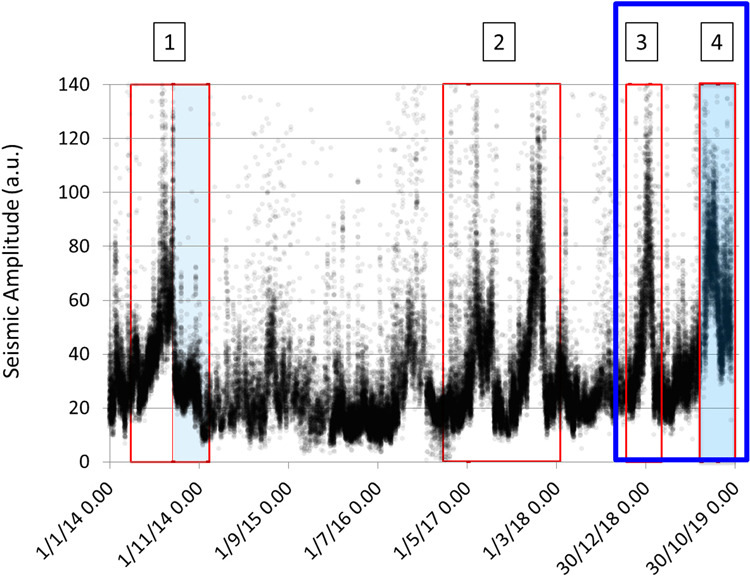
Seismic amplitude from January 1, 2014 to September 15, 2019. The time series consist of signal intervals of three different stations (STRA, STRE and STR1), properly normalized to avoid time gaps. The highlighted periods refer to: 1) crisis of 2014; 2) 2017–2018 reawakening phase; 3) November 2018-January 2019 increase of the activity; 4) phase of the paroxysms of July and August 2019. The effusive phases are highlighted in light blue. The box with the blue outline indicates the period analyzed in this work.

Generally, all the seismic signals related to volcanic dynamics (explosion-quakes, volcanic tremor, landslide signals, spattering, puffing, etc.) increase in periods when the eruptive activity intensifies^25,28^. For this reason, the seismic amplitude, here expressed as the mean square of the 3-component signal module, is a very robust parameter to represent the level of activity of Stromboli. Figure 4 consists of a collage of different station data to overcome gaps in the time series of individual stations, and shows four periods of seismic amplitude increase corresponding to: 1) the 2014 crisis, which culminated in the August-November effusive eruption^28,29,42^ 2) the 2017-2018 reawakening phase^30^, which was characterized by the resumption of major explosions and lava overflows from the summit vents and led to the raising of the alert level from green to yellow; 3) the 2018-2019 increase of eruptive activity, which again led to change the alert level from green to yellow, without culminating in remarkable eruptive activity anomalies; 4) the summer 2019 eruptive phase, which began with the July 3 paroxysm (Fig. 2a) and was characterized by effusive activity and by a second paroxysm occurred on August 28 (Fig. 2b).

The last episode (4 in Fig. 4) is peculiar because a clear increase in seismic amplitude occurred only after the paroxysm of July 3. This means that in this case, in contrast to what was reported for episodes 1, 2, and 3 (in Fig. 4), the seismic amplitude was not a precursor of abnormal eruptive activity. Therefore, we analyzed the seismic data focusing our attention not only on the signal amplitude and explosion-quake occurrence rate that are routinely monitored^30^, but also on other characteristics of the seismic signals. As mentioned above, we consider for our analysis the period November 15, 2018 - September 15, 2019, when the STRA station, the closest to the eruptive vents, was continuatively active. This period includes phases 3 and 4 of Fig. 4 (box with the blue outline in Fig. 4).

### VLP size

**Figure 5:**
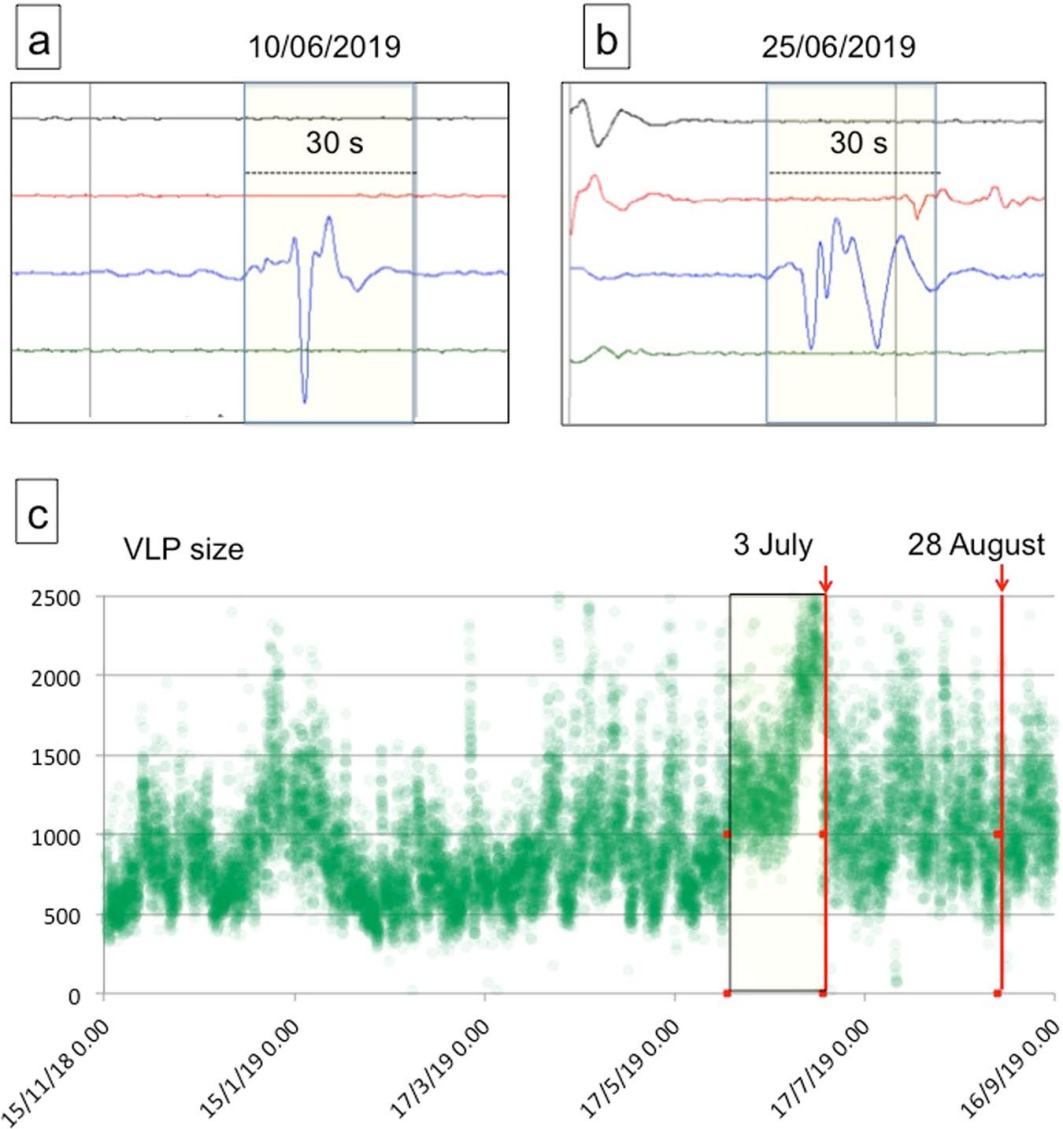
Comparison of VLP waveform recorded by the STRA station, E-W component. (**a**) one event characterized by a single pulse; (**b**) one event characterized by prolonged oscillation; (**c**) time series of VLP size from November 15, 2018 to September 15, 2019 (amplitude in counts). Panels a and b are details of a 4-hour seismogram plot. The time difference between the beginning of each line is 10 minutes. The VLP event in panel a) was registered at 20:24 on 10 June whereas the VLP in panel b) was registered at 21:40 on 25 June 2019. The yellow rectangle indicates the period between the beginning of the variation (June 2) and the first paroxysm (July 3).

We have exploited the typical seismic signals of the Strombolian activity, which are the VLP events^35^ to define a parameter that depends on the waveform, in terms of amplitude and duration of the VLPs. Observing the VLP (0.05-0.5 Hz frequency band) component of the seismic signal before the paroxysm of July 3, 2019, variations in the waveform that precede the paroxysm can be noticed (Fig. 5a,b). The waveform reported in Fig. 5b, characterized by a prolonged oscillation with respect to that reported in Fig. 5a, which shows a single pulse of greater amplitude, becomes more and more frequent before the July 3 paroxysm.

To exploit this feature we introduced a new parameter named *VLP size* (Fig. 5c). This parameter is given by the maximum RSAM value of 1770 windows (30-second duration) of bandpass filtered signal (0.05 − 0.5 Hz frequency band) calculated on sliding windows that move by 1 second, covering an interval of 1800 seconds (30 minutes) (see Methods). The calculation of *VLP size* gives one value for each half hour. This parameter, in its current formulation, is suitable for characterizing the Strombolian VLP seismicity and is not a general method to characterize seismic signals. We used the E-W component of the STRA station that is radial with respect to the typical position of the VLP sources (Fig. 3e). Generally, events with prolonged oscillation (Fig. 5b) have a larger VLP size than those with a single pulse (Fig. 5a). The VLP size shows a clear increase in the period preceding the July 3 paroxysm, starting from June 2.

### Polarization analysis

**Figure 6:**
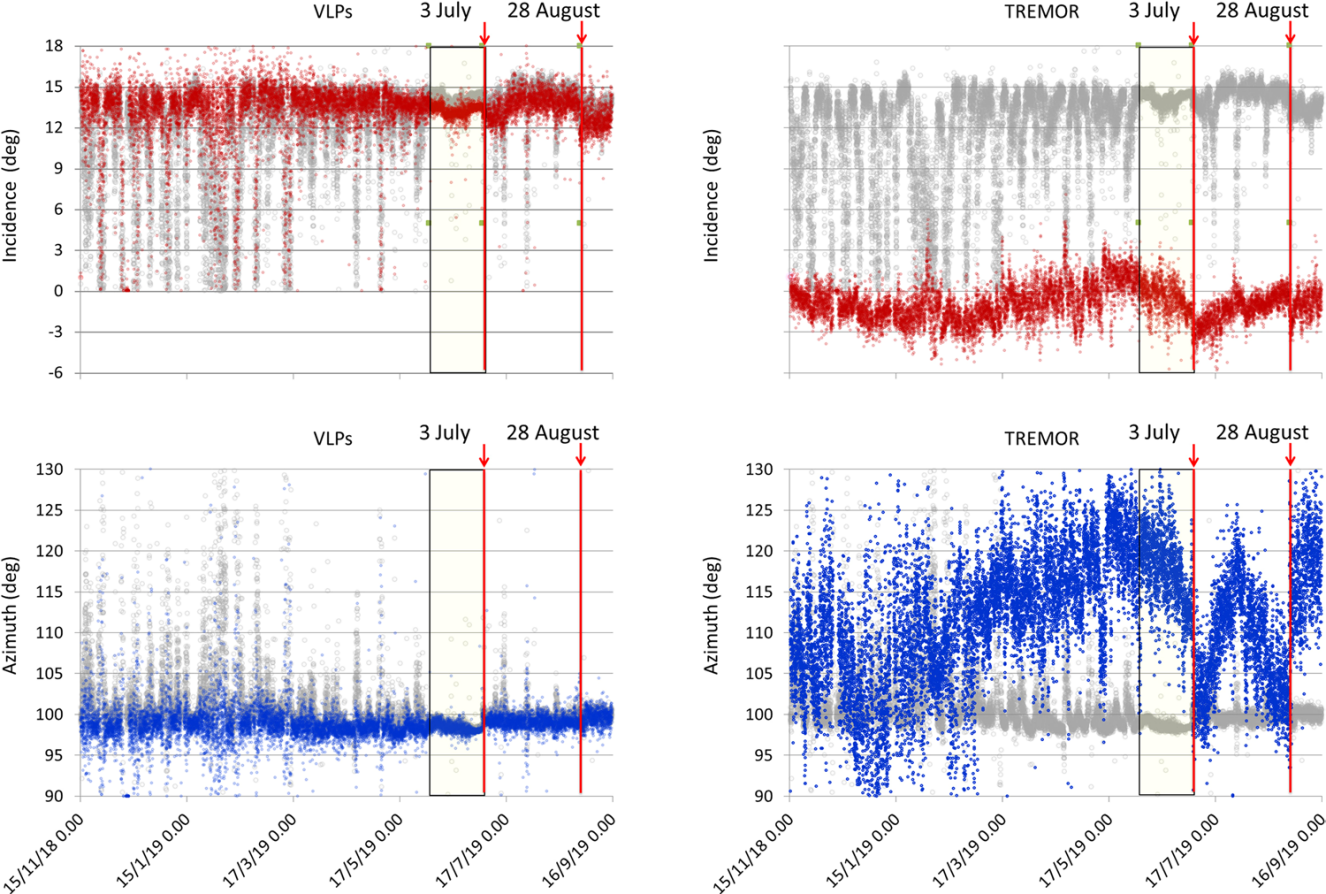
Comparison between the polarization parameters of the unfiltered signal (gray dots) and the polarization parameters (blue dots for azimuth and red dots for incidence angles) of the tremor (on the right) and VLP (on the left) frequency bands, 1–3 Hz and 0.05–0.5 Hz respectively. The azimuth is represented in degrees from north to east. The incidence is in degrees with respect to the horizontal. The position of the STRA station is shown in Fig. 1. The yellow rectangles indicate the period between the beginning of the variation (June 2) and the first paroxysm (July 3).

The signals generated by the explosions and the continuous volcanic tremor dominate the seismic wave field of Stromboli^24,34–36^ and are typically near horizontally polarized. The polarization analysis^44^ of the signal recorded by the 3-component STRA station shows modest changes in the period preceding the July 3 paroxysm (Fig. 6). In addition to the modest variation of the azimuth angle, another interesting feature was reported between June 1 and July 3, 2019. As it can be noticed, the signal is more focused, and the azimuth and incidence angle dispersion, which is evident in the previous period (November 15, 2018 - May 31, 2019), disappears. We repeated the polarization analysis in the tremor (1-3 Hz) and VLP (0.05 – 0.5 Hz) frequency bands to investigate whether the signal focusing was due to a specific source (Fig. 6). Comparing the results with the time series of the unfiltered signal (gray dots in Fig. 6), we note that the signal polarization is dominated by the VLP component and the signal focusing can be explained by an increased contribution of the VLP component in the seismic wave field. On the other hand, the polarization of tremor frequency band shows remarkable variations in the period preceding the July 3 and August 28 paroxysms (Fig. 6).

**Figure 7:**
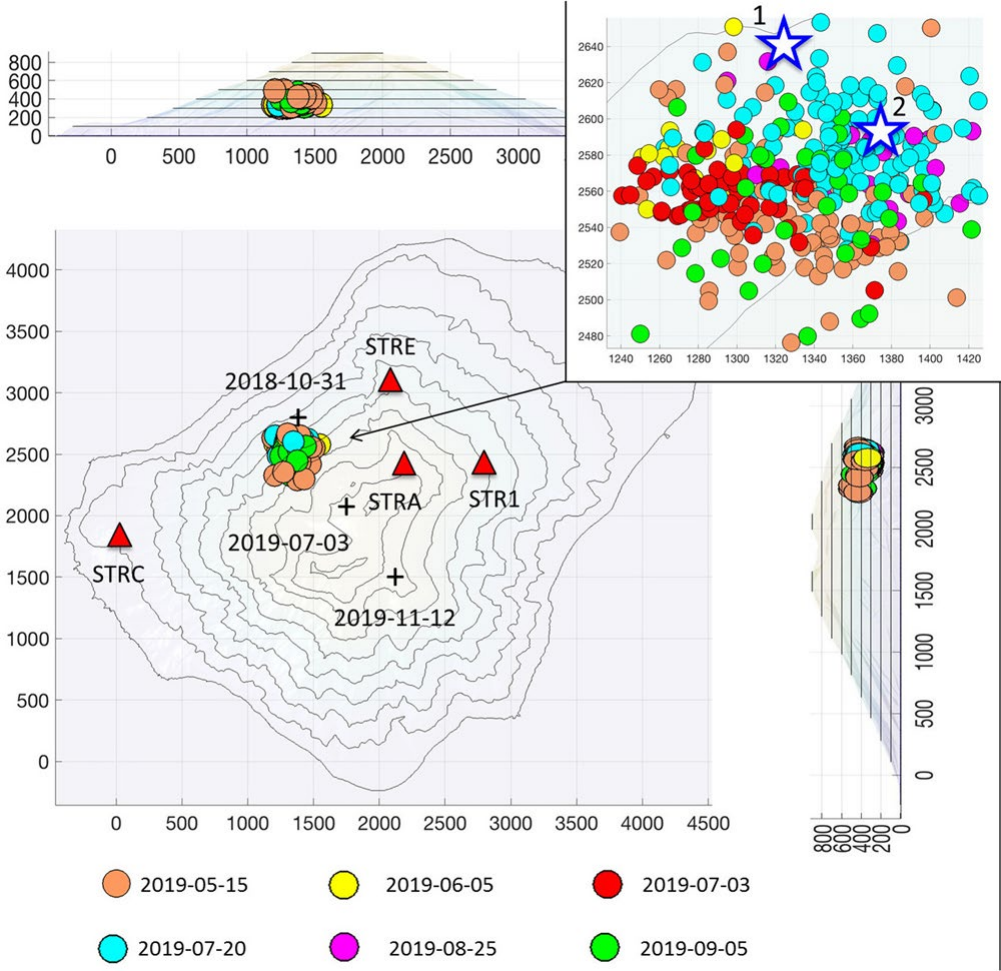
Locations of 360 selected VLP events recorded between May and August 2019. The colors indicate the date of the events. The distribution of VLPs over the different days is as follows: 94 on May 15th; 18 on June 5; 59 on July 3 (all before the paroxysm); 127 on July 20; 22 on August 25 and 40 on September 5. The black crosses indicate the epicenters of three of the four VT events recorded in the period of interest. The labels show the date in a yyyy-mm-dd format. A fourth event, which occurred on June 13, 2019, falls slightly off the map, towards northwest. Table 1 shows the locations of all the VT events. The blue stars in the upper right map represent the location of the major explosions occurred on June 25 (1) and August 29 (2), 2019. The software used to create the map is Matlab R2017a (https://it.mathworks.com/products/new_products/release2017a.html).

We exploited the radial polarization of VLPs with respect to the source to locate 360 selected events occurred between May and September 2019 (Fig. 7), which were well recorded at four stations (STR1, STRA, STRC and STRE). The locations do not show remarkable variations before and during the eruptive phase of the summer 2019. However, we can recognize a greater concentration of the VLP sources towards south-west in the period before the July 3 paroxysm (yellow, orange and red dots in Fig. 7), while most of the sources on July 20 and August 25 are concentrated towards north-east (cyan and magenta dots in Fig. 7). Those of September are slightly more dispersed (green dots in Fig. 7).

The stars in the map at the top right of Fig. 7 indicate the position of the source of two major explosions recorded on June 25 (star 1: UTM 518044 E, 4294249 N; elevation 230 m asl) and August 29 (star 2: UTM 518095 E, 4294195 N; elevation 354 m a.s.l.), evaluated by using the polarization parameters, as described in Methods section, for the locations of the ordinary VLPs.

Furthermore, we estimated the location of four volcano-tectonic earthquakes (VT), which occurred in the period of interest, using the NLLoc program^45^. We report in Table 1, the locations of the VT events. Crosses in Fig. 7 mark three of the VT epicenter locations.

**Table 1:** Hypocentral parameters of the VT seismic events recorded in Stromboli during the period of interest.

ORIGIN TIME (dd/mm/yyyy hh:mm)	LATITUDE	LONGITUDE	DEPTH (km)	STATIONS (used for localization)
31/10/2018 15:27	38°N 47.87’	15°E 12.45’	4.10	STRG, STRC, STR3, STRE, SVO
13/06/2019 13:19	38°N 48.67’	15°E 10.56’	4.10	STRG, STRE, STRA, STR1, STR4, SVO
03/07/2019 14:44	38°N 47.51’	15°E 12.77’	0.77	STRC, STRG, STR1, STR3, STR4, SVO
12/11/2019 03:11	38°N 47.20’	15°E 13.02’	1.52	STR4, STR1, STRA, STR3, STRE, SVO

### Fractal dimension analysis (FD)

**Figure 8:**
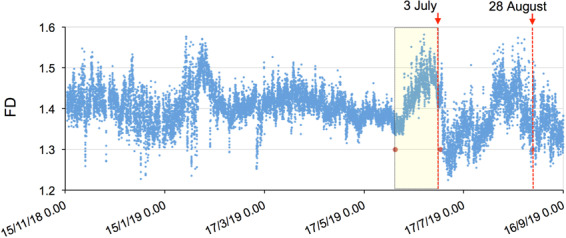
Time evolution of the Fractal Dimension (FD) of the STRA station unfiltered seismic signal. The FD is calculated on 30-minute contiguous windows from November 15, 2018 to September 15, 2019. The yellow box highlights the FD change before the July 3 paroxysm. The yellow rectangle indicates the period between June 2 and July 3.

We evaluated the Fractal Dimension of the STRA (E-W component) in order to detect precursors of the paroxysmal activity. Time-varying FD analysis has been conducted during eruptive episodes on active volcanoes^46–51^ as a means of studying their dynamics. The FD provides significant features that describe the complexity of the volcanic system, and their time variations allow the detection of subtle changes that can be interpreted in terms of short-term precursors of eruptive activity^50,51^. The evolution of the FD is shown in Fig. 8.

The FD fluctuated during the studied period with an average value FD = 1.4. FD reached values above average on early February, before July 3 and before August 28, and below average after July 3 paroxysm (Fig. 8). The change before the July 3 paroxysm started with a significant increase on June 5 followed by an abrupt decrease that started on June 25.

### Paroxysm data analysis

**Figure 9:**
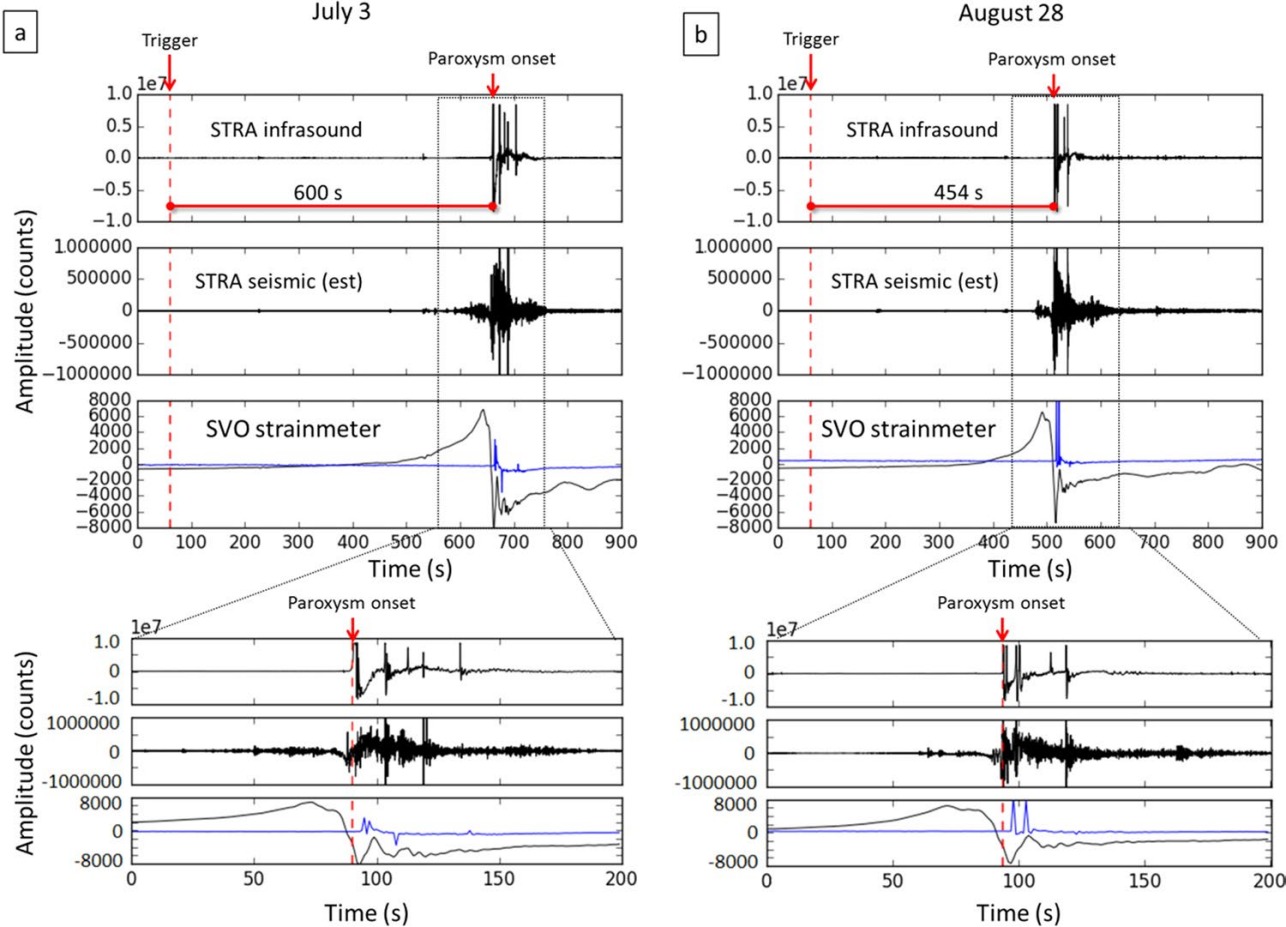
Automatic triggers of the strainmeter signals of the July 3 (**a**) and August 28 (**b**) paroxysms. The blue curve superimposed on the strainmeter data represents the barometric pressure. At the bottom, zooms of the infrasound, seismic and strainmeter signals of both the paroxysms.

We mark the onset of the July 3 and August 28 paroxysmal events using the signal recorded by the infrasonic sensor at STRA station, located about 550 m from the eruptive vent. By comparing the infrasonic and the seismic signals of the STRA station, we can recognize an increase in the amplitude of the seismic signal about two minutes before the onset of the July 3 paroxysm, due to a sequence of explosions, close in time, and to an intense spattering activity (Fig. 9a). For the August 28 event, changes in the seismic signal can be recognized about one minute before the onset of the paroxysm (Fig. 9b). Considering the data of the SVO strainmeter, it can be seen that there is a strain variation minutes before the onset of the paroxysmal explosions. Following the approach proposed in^30^ and in^8^ based on an appropriately tuned Short-Term Averaging/Long-Term Averaging (STA/LTA) algorithm^43,52^ (see Methods section), we performed automatic triggers of the July 3 and August 28 paroxysm strainmeter signals by using the STA/LTA routines of “ObsPy” data analysis system^44^. We obtained a trigger 10 minutes (600 s) before the onset of the July 3 paroxysm (Fig. 9a) and about 7.5 minutes (454 s) before the onset of the August 28 paroxysm (Fig. 9b).

**Figure 10:**
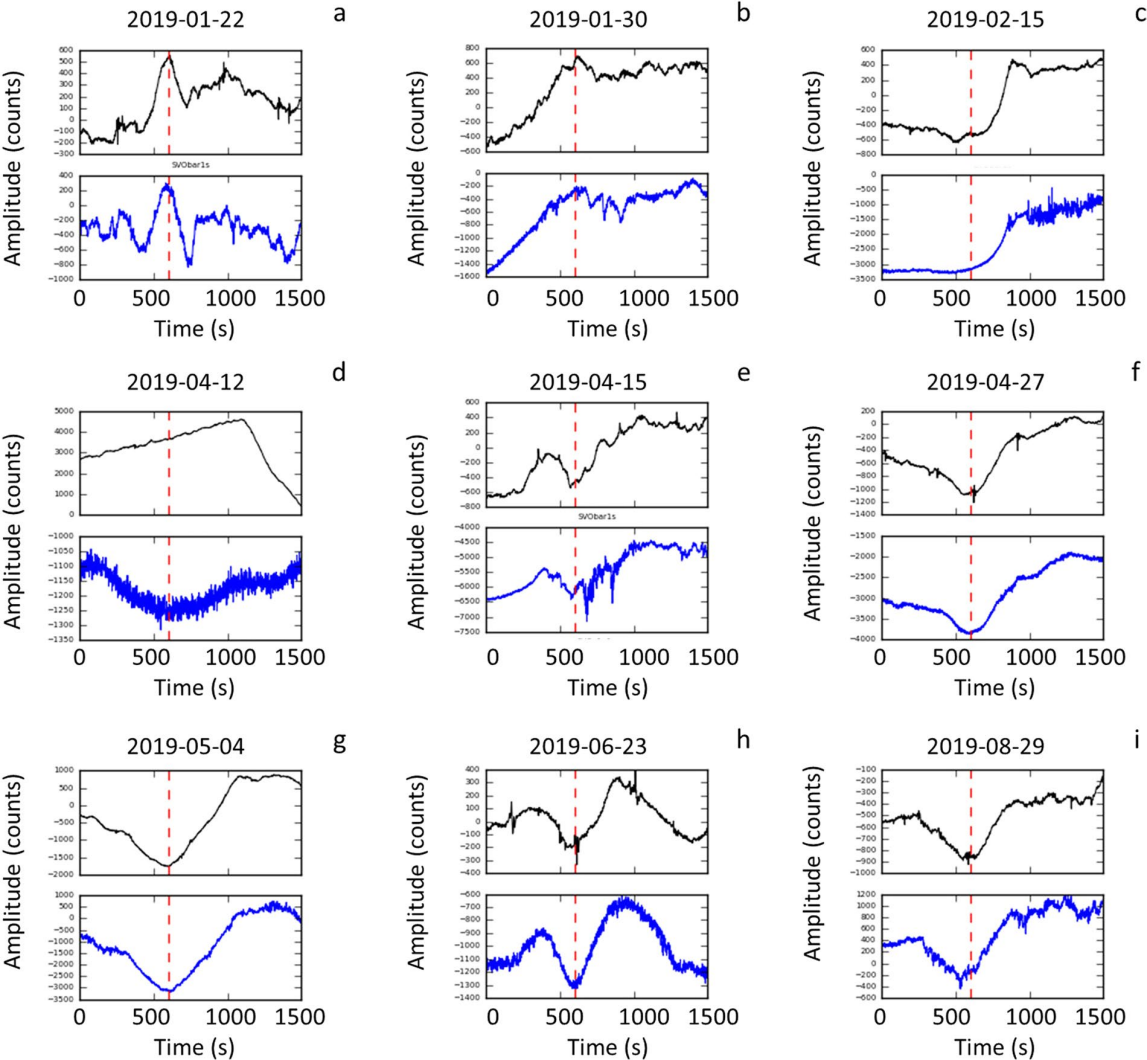
Nine of the eleven automatic triggers not corresponding to paroxysms. The black curve is the SVO strainmeter signal (top panel of each plot). The blue curve is the atmospheric pressure measured at the strainmeter’s wellhead. Two further triggers were detected on November 16, 2018 and June 2, 2019, not shown in the figure. The trigger of April 12, 2019 (d) is the only one in which no correlation is observed between the barometric signal and the strainmeter signal.

We also applied the STA/LTA algorithm on the SVO strainmeter time series from November 15, 2018 to September 15, 2019 and we obtained 11 triggers not associated with paroxysms. Analyzing the triggers in relation to the atmospheric pressure measured at the strainmeter’s wellhead, it can be noticed that, in general, the barometric pressure correlates with the strainmeter signal in triggers not associated with paroxysmal explosions (Fig. 10), whereas for the July 3 and August 28 paroxysms the atmospheric pressure and strainmeter signal are independent of each other (Fig. 9). Only one (Fig. 10d) of the 11 triggers not associated with paroxysms shows the barometric and strain signals independent of each other. This abnormal strainmeter signal was reported in the INGV surveillance bulletins (http://www.ct.ingv.it).

**Figure 11:**
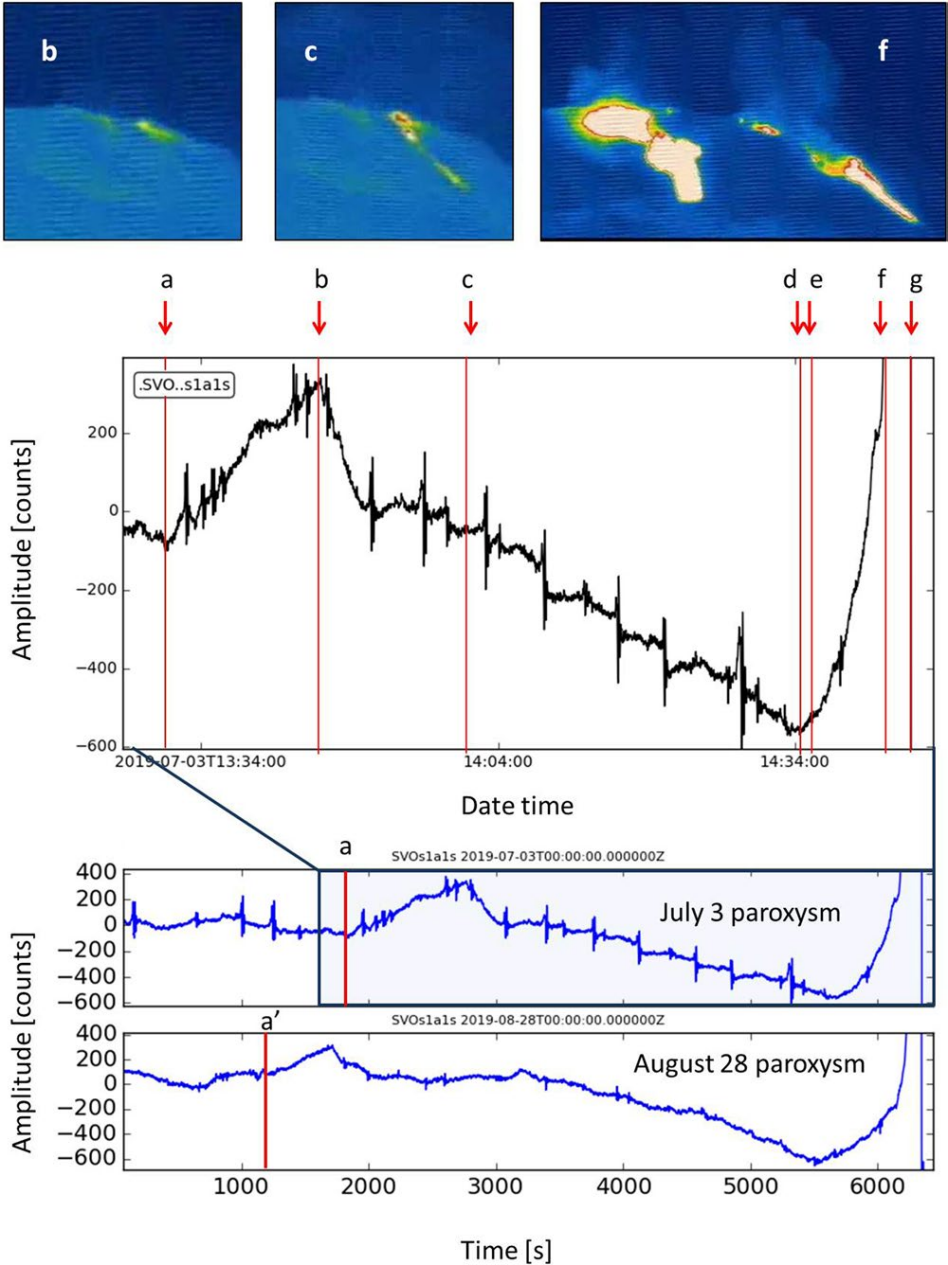
Signals of the SVO strainmeter recorded before the July 3 and August 28, 2019 paroxysms. The magnified signal relating to the July 3 (top of the plot) is compared with the precursory effusive phase recorded by the cameras. (**a**) is the beginning of a strain increase (13:30:00 UTC); (**b**) marks the first minor lava overflows (13:46:00); (**c**) indicates a small vent opening at the base of NE crater, feeding a thin lava flow (14:00:30); (**d**) minimum strain (14:34:32); (**e**) automatic trigger (14:35:44); (**f**) small intra-crater lava flow (14:43:10); (**g**) paroxysmal explosion beginning (14:45:40). The INGV camera images at the top of the figure indicate the moments relative to the corresponding letters. *b* and *c* are camera images from the SQT camera (view from NE of the NE crater flank) and *f* camera image is from the SPT camera (view from South of the whole crater terrace). SQT and SPT are Q and P in Fig. 1, respectively. The software used to create the figure is Python 2.7 (https://www.python.org/).

Furthermore, the analysis of the images recorded by the INGV camera monitoring network allowed us to identify effusive phenomena anticipating the July 3 paroxysm and to obtain their timing. The camera images displayed minor lava overflows from the NE crater that started around 13:46:00 UTC (*b* in Fig. 11), feeding a thin lava flow, which lasted ~ 43 minutes. In the meanwhile, at 14:43:10 (*f* in Fig. 11) other small lava flows started simultaneously from at least two vents in the central crater area. We compared the timing of this precursory effusive phase with the signal of the SVO strainmeter (Fig. 11). We found that the beginning of the first minor lava overflows (*b* in Fig. 11) co-occurred with the beginning of a strain decrease phase that lasted until 14:34:32 (*d* in Fig. 11), when the signal suddenly changed and showed a significant strain increase. About one minute later, at 14:35:44 we obtain the automatic trigger with the STA/LTA algorithm (*e* in Fig. 11). The letter *f* in Fig. 11 marks the beginning of the small intra-crater lava flows that occurred at 14:43:10. Two and a half minutes later the paroxysmal explosion began (*g* in Fig. 11), as recorded by the STRA infrasonic sensor.

A temporal evolution of the strainmeter signal similar to the one described above, which we observed before the July 3 paroxysm, is recognizable also in the period preceding the August 28 paroxysm (Fig. 11) for which, unfortunately, we have no useful information from the cameras, both because a significant effusive phase was in progress and because the activity was concentrated on the southern side of the “Sciara del Fuoco” slope, out of the cameras field of view. For both the paroxysms, before the strain decrease phase, which began at time “*b*” (Fig. 11) for the July 3 paroxysm, there was a strain increase, which for the July 3 event started at 13:30:00 (*a* in Fig. 11). The described temporal evolution of the strainmeter signal can be interpreted as the effect of an overpressure source at depth that caused the ascent of the magma column inside the conduit accompanied by an increase in the strain (from *a* to *b* in Fig. 11).

## Discussion

**Figure 12:**
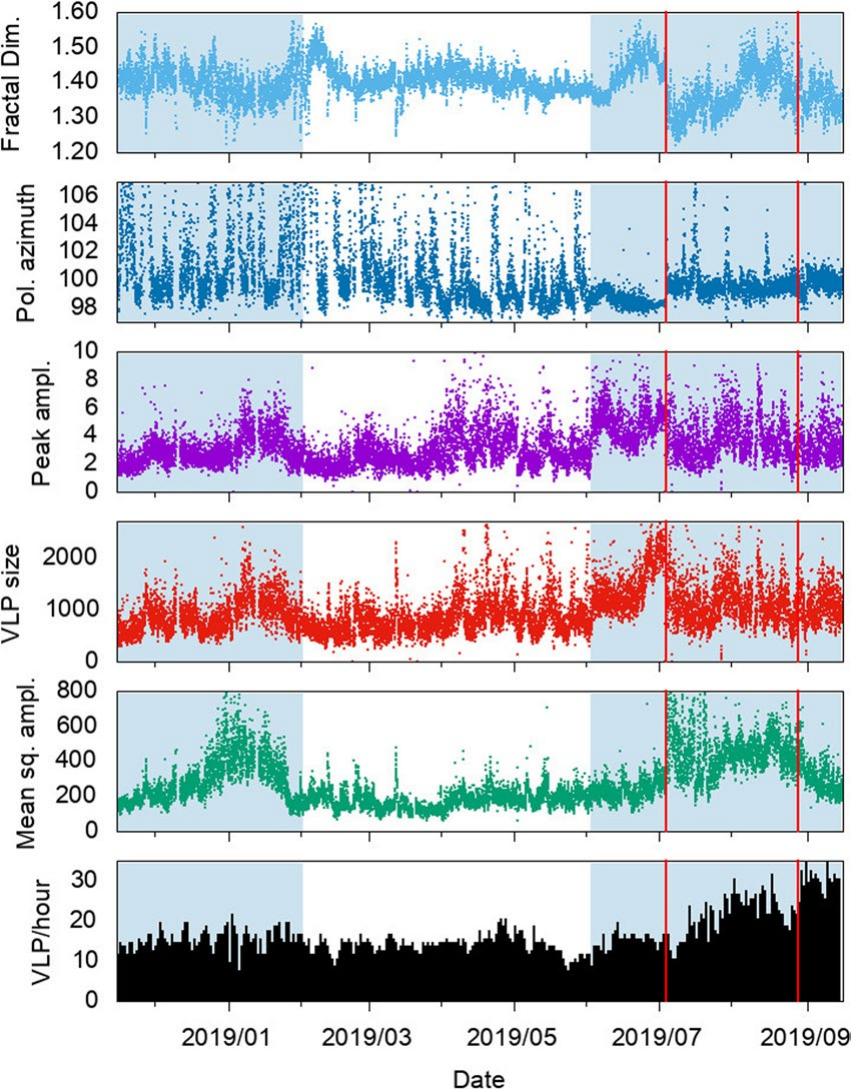
Comparison of the time series of routinely monitored seismic parameters with the parameters calculated in this article, in the period November 15, 2018 - September 15, 2019. VLP / hour is the daily VLP hourly rate; Mean sq. ampl. is the Mean Square Amplitude of the 3-component signal module of the STRA station; VLP size is calculated for STRA East component, which is radial with respect to the source position of the VLPs; Peak ampl. is the VLP peak-to-peak amplitude calculated on the STRA East component; Pol. azimuth is the azimuth of the polarization of the unfiltered STRA signal; Fractal Dim. is the fractal dimension of STRA East component. The blue rectangles highlight the period between 15 November 2018 and 31 January 2019, related to the November 2018-January 2019 increase of the activity (episode 3 in Fig. 4) and the period between June 2 (beginning of the variation of most parameters) and September 15. The time series shown in this figure are available as Supplementary Data.

The eruptive phase of July-August 2019 was the most serious volcanic crisis at Stromboli in the last decades and resulted in a fatality and some injuries. The above-described analysis allowed us to recognize changes in Stromboli’s activity from about one month before the July 3 paroxysm that the routinely monitored parameters did not allow to detect. Figure 12 shows the VLP hourly rate and the seismic amplitude, which are routinely monitored, compared with the results of the time series analysis described in the previous section. We have also included in Fig. 12 the time series of the peak-to-peak amplitude of the VLP events that was calculated over the same time interval. Comparing the July-August 2019 (4 in Fig. 4) with the November 2018 – January 2019 (3 in Fig. 4) eruptive phases, the last of which did not culminate in abnormal eruptive activity such as lava flows or paroxysms, we noted that both of them show variations (Fig. 12), but the evolution of the parameters was different in the two periods. In particular, in the November 2018 – January 2019 eruptive phase there was a significant increase in the seismic amplitude followed by a moderate increase in the VLP size and VLP peak-to-peak amplitude. Conversely, no significant changes of seismic amplitude were detected before the beginning of the July- August 2019 eruptive crisis, but the VLP size and VLP peak-to-peak amplitude increased significantly from about a month before the July 3 paroxysm. The polarization parameters did not show significant variations in the November 2018 – January 2019 phase, whereas showed modest changes before the July-August 2019 eruptive phase. In particular, the polarization azimuth (Fig. 12) was focused in a narrow interval of about two degrees (98°−100° N) indicating a greater contribution of the VLP radiation to the seismic wave field (Fig. 12). It is worth noting that the polarization azimuth of the STRA signal filtered in the tremor frequency band (1–3 Hz) in the period before the July 3 paroxysm shows significant variations as well as in the period preceding the August 28 paroxysm (Fig. 6). This could be related to changes of activity in the vent areas (NE, C, SW in Fig. 1). This observation can be exploited to highlight anomalous changes in the activity in the crater area possibly related to an impending paroxysm.

Regarding the fractal dimension (top plot in Fig. 12), we find that it decreased in the November-December 2018 period, consistently with the intensification of the eruptive activity. On the other hand, in the period before the July 3 paroxysm, the FD increased significantly, suggesting a greater contribution of the VLP component (see Methods), consistently with the temporal evolution of the VLP size and the VLP peak-to-peak amplitude.

For what concerns the short-term analysis, the strainmeter data indicates a promising possibility to realize an early warning system for paroxysmal explosions. In general, automatic systems^55^, are based on a detection task (e.g. a trigger algorithm), a validation task and one or more tasks for analysis and notifications, which can be used to undertake appropriate actions. In the case of Stromboli paroxysms, the use of a properly configured STA/LTA algorithm is suitable to trigger sudden and significant strain changes (detection task), such as those that precede the paroxysmal explosions by about 7.5–10 minutes. Such sudden changes in the strain can also be caused by abrupt variations in atmospheric pressure. For this reason the strainmeter is equipped with a wellhead barometer. The comparison between strainmeter data and barometric data allowed us to discover that 10 of the 13 triggers obtained with the STA/LTA algorithm were associated with changes in the barometric pressure, 2 of them with paroxysms, whereas 1 remains unexplained. In future developments, the correlation between the strainmeter signal and the barometric signal will be used to set up a validation criterion (validation task) to automatically discard the triggers due to sudden changes in atmospheric pressure. We believe this is a promising approach to set up a timely warning automatic system for the detection of a strain precursor that anticipates a sudden and highly dangerous event such as paroxysm by nearly 10 minutes. We recall in this regard that the paroxysm of July 3, 2019 caused the death of a person who was near the southern edge of the Sciara del Fuoco. A 7.5–10-minute notice can be a sufficient time to take actions for the safety of people on the Island, allowing them enough time to reach safe places that must of course be previously identified with respect to the impact of a paroxysm.

Concerning the dynamics of paroxysmal explosions at Stromboli, a general model is not yet available and remains a matter of debate^22,23,56^. However, it is known that the shallow part of the Stromboli conduit is filled by gas-poor and high porphyricity magma (HP), which is ejected during the ordinary Strombolian explosions, whereas low porphyricity (LP) and gas-rich magma fills the conduit at a greater depth. This type of magma is emitted during paroxysms. This is why it is generally accepted that paroxysmal explosions are triggered by the fast rise of low porphyricity (LP) and gas-rich magma batches from the storage zone located at 5–10 km depth^13,14,57–60^. The fast rise of this gas-rich LP magma causes inflation and oscillation of the upper conduit^13,61^. It has also been found that the gases emitted during paroxysmal events have different chemical compositions from those released during ordinary Strombolian explosions, with paroxysms containing much higher amounts of SO_2_ and CO_2_^21^. An additional aspect that emerged from the observations of the July 3 paroxysm was the progressive moderate decrease of the strain before the explosion, accompanied by a lava overflow, which lasted about 43 minutes (from *b* to *d* in Fig. 11). In our conceptual model, we interpret the strain decrease phase, which accompanied the lava overflow, as an effect of the gradual filling of the upper conduit with low-density (LP) gas-rich magma just before the triggering of the paroxysm. A similar behavior was observed also before the August 28 paroxysm (Fig. 11) and could be exploited to obtain an earlier detection of the paroxysm precursor (e.g about 1 hour before the explosion), in future developments.

The seismic data recorded before the July-August 2019 paroxysmal eruptive phase allowed us to highlight significant changes in the parameters related to the VLP event dynamics, that are due to the gas slug migration inside the conduit, as proven by several studies^35,62–64^. The changes of the VLP dynamics before the Summer 2019 eruptive phase, highlighted by the VLP size, VLP peak-to-peak amplitude and FD, are not related to the source location of VLPs (Fig. 7) or to the occurrence rate (Fig. 12), which do not change significantly. They are instead linked to the temporal evolution of the explosive source, that results in the waveform of the VLPs^53,54^. These changes indicate higher gas content in the Strombolian explosive activity, starting at least one month before the July 3 paroxysm. Our analyses suggest a critical role of the gas as well as of the coexistence of two magma-types, HP and LP magma, for the eruptive dynamics of Stromboli. Consequently, the *VLP size* and peak-to-peak amplitude are sensitive to these changes and can be considered as medium-term precursors of the paroxysm of July 3. This study provides some interesting correlations that should motivate future work at Stromboli and other volcanoes where VLPs are observed.

## Methods

At the end of 2018, the seismic network of the Stromboli island was composed by 8 stations (Table 2) managed by Osservatorio Vesuviano (INGV-OV) and Osservatorio Etneo (INGV-OE), which are deployed as shown in Fig. 1. The seismic stations are equipped with Guralp CMG40T 60s-50Hz velocimeters with sensitivity of 800 V/m/s. Data are acquired using GILDA^65^ or GAIA digitizers^66^. The data transmission is realized by UHF digital radio links and thorough the INGV WiFi data infrastructure^66,67^. The data are received by the high availability systems^68^ of the local acquisition centers in Stromboli and Lipari and send to the acquisition centers in Naples (INGV-OV) and Catania (INGV-OE), in real time.

**Table 2:** Technical characteristics of the seismic network stations.

Station	Sensor	Datalogger	Sampling rate sps
STR1	Guralp CMG40T	GAIA	50
STR4	Guralp CMG40T	GILDA	50
STRE	Guralp CMG40T	GAIA	50
STRC	Guralp CMG40T	GILDA	50
STRG	Guralp CMG40T	GILDA	50
STRA	Guralp CMG40T	GAIA	50
STR3 (renamed IST3)	Nanometrics Trillium120PA	Nanometrics Trident	100
SVO	accelerometer	GILDA	100

STRA and STRG stations are also equipped with infrasonic sensors (Chaparral Model 25). STRC and STRE were destroyed by the fire caused by the July 3 paroxysm and were reinstalled 10 days later. Station STR4 was burn on July 31, 2019 and reinstalled on October 30, 2019.

In our analysis, we defined the VLP size that is a parameter sensitive to the waveform changes of VLPs due to the ordinary explosive activity. The *VLP size* is based on the RSAM function defined as follows^43^:

$$VLP\,size=\frac{1}{T}\mathop{\sum }\limits_{t=iT\frac{T}{2}}^{iT+\frac{T}{2}}|s(t)|$$

Where T is the time interval and s(t) is the seismic signal. To obtain the VLP size we filtered the signal E-W component of the STRA station in the VLP frequency band (0.05–0.5 Hz) and divided it into half-hour windows (1800 seconds each window). Then we calculated the RSAM of a 30-second sliding window shifted by 1 second. The 30-second duration of the sliding window was chosen through a trial and error approach to obtain a good sensitivity to the VLP waveform variations. We choose as VLP size the maximum of the 1770 values obtained for each half-hour window, which corresponds to the size of “the largest” VLP event in that half-hour interval. Thus, we obtain the *VLP size* time series from November 15, 2018 to September 15, 2019 shown in Fig. 5c. We adopted a similar approach to estimate the VLP peak-to-peak amplitude (Fig. 11), dividing the VLP filtered signal into half-hour windows and considering a 30-second sliding window with a 1-second shift. For each 30-second window we calculated the sum of the minimum and maximum absolute values, then we chose the maximum of these values as the VLP peak-to-peak amplitude. Also in this case the chosen parameter is the maximum VLP peak-to-peak amplitude in a half-hour interval.

To investigate the polarization of the seismic signals we used Obspy tools^44^ based on the singular value decomposition of the covariance matrix of the 3-component seismic signal (vertical, E-W, N-S). For the long-term time series (Figs. 6, 12), we performed the polarization analysis of the data recorded by the 3-component STRA station. We carried out the polarization analysis of 30-minute contiguous windows of the raw signal (gray dots in Fig. 6) and of the signal filtered in the tremor frequency band (1–3 Hz) (right plots in Fig. 6). Furthermore, we performed the polarization analysis of the VLPs identified with the *VLP size* analysis (the largest event of each half-hour signal window), whose results are shown in the left plots of Fig. 6.

For the location of the VLPs we performed the polarization analysis of a set of 360 VLP events recorded by four 3-component seismic stations that had a good functioning in the period of interest (STRA, STRE, STRC and STR1). We found the location of the VLP events exploiting their typical radial polarization towards the source (Fig. 7). Through polarization analysis^44^, we obtained the estimation of the eigenvectors of the 3-component covariance matrix of the VLP signals at the four selected stations, which allowed us to know the direction of polarization defined by the azimuth and incidence angles for each VLP event. We developed an algorithm to calculate the point of minimum distance between the polarization directions for each VLP event. We estimated an uncertainty on the locations ranging between 250 and 350 m.

The seismic amplitude (Figs. 4, 12) is routinely calculated as the mean square of the 3-component module of contiguous half-hour signal windows recorded by STRA station.

We performed the time-varying Fractal Dimension analysis (FD) applied to the seismic signal recorded by the STRA station (E-W component). We calculated the FD time evolution applying the Higuchi algorithm^69^, using *k* = 6 and *N* = 90,000 samples (30 minutes) with no window overlap. The angular coefficient of the linear regression of the graph *log(L(k*)) *vs. log(1⁄k)* provided the FD, where *log* is the natural logarithm.

The Higuchi algorithm^69^ generates multiple time series from *N* equal-spaced sampled signal (*x(i), i* = *1,…,N*), creating new time series ${x}_{k}^{m}$ as follows:

$${x}_{k}^{m};x(m),x(m+k),x(m+2k),\mathrm{...}.,x(m+[\frac{N-m}{k}].k)(m=1,2,\mathrm{...}.,k),$$

with *m* and *k* integers and [a] denoting the integer part of a.

For each time series, the absolute differences between each two successive data points are summed to calculate the vertical length of the signal with the scale size *k* as follows:

$${L}_{m}(k)=\left\{(\mathop{\sum }\limits_{i=1}^{\left[\frac{N-m}{k}\right]}|x(m+ik)-x(m+(i-1)k)|)\frac{N-1}{\left[\frac{N-m}{k}\right].k}\right\}$$

The length of the series segment *L(k)* is the mean of the *L*_*m*_*(k)* values,

$$L(k)=\frac{{\sum }_{m=1}^{k}{L}_{m}(k)}{k},for\,m=1,\ldots .,k.$$

When *L(k)* is proportional to ${k}^{-D}$, then the signal is fractal-like and has the fractal dimension *D*.

**Figure 13:**
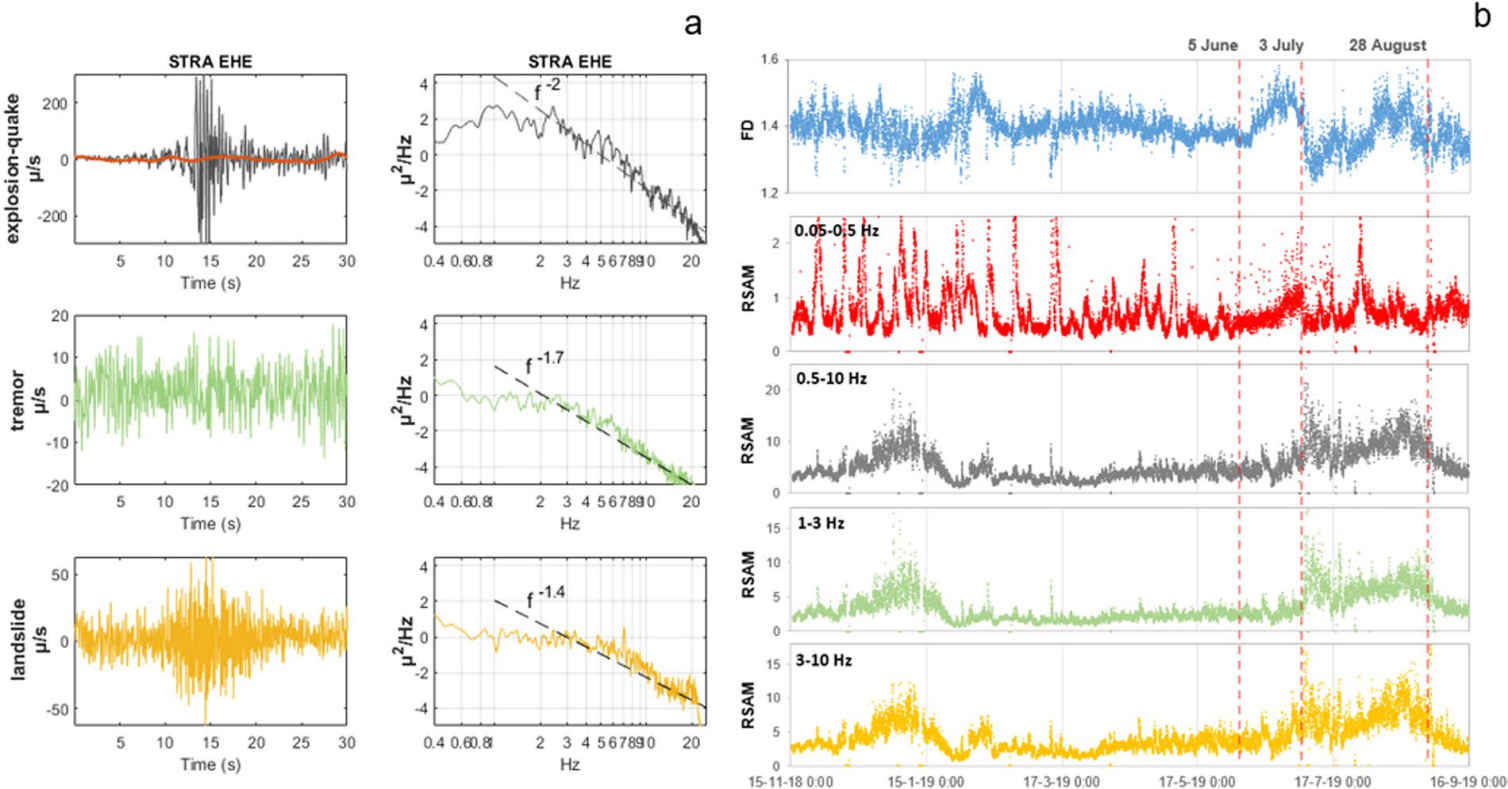
(**a**) Examples of an explosion-quake in dark-gray with the VLP content highlighted in red, tremor in green and landslide in yellow with the corresponding PSD curves including fitted linear trends on high frequencies (1–25 Hz). (**b**) Fractal dimension and RSAM (µ/s) for different frequency bands. The software used to create the figure is Matlab R2013a (https://es.mathworks.com/products/matlab.html).

Concerning the interpretation of the FD (top plot in Fig. 12), we studied examples of explosion-quake, tremor and landslide events (same as used in Fig. 3), calculating their Power Spectra Density (PSD) and fitting a linear curve to the high frequencies (from 1 Hz to Nyquist=25 Hz). The highest-degree frequency decay corresponds to the explosion-quake signal (*f* ^*– 2*^*)* and the lowest to the landslide event *(f* ^*−1.4*^*)*, as can be seen in Fig. 13a. Higuchi^69^ showed that a curve with a single power-law spectrum is self-similar and the index, *α*, of its PSD, has a power-law dependence on frequency *P(f) ~ f* ^*- α*^, and is related to the fractal dimension *D* by the equation *D = (5- α)/2 (*for 1 < *D* < *2)*. Therefore, we can link the source spectra of the different signals included in the seismic record of STRA station with different values of FD. In order to highlighting the contribution of the different sources, we applied the Real-Time Seismic Amplitude Measurement (RSAM)^70^ methodology to STRA data. Figure 13b, shows RSAM (in µ/s) curves for the different filtered-band channels: very low frequencies (0.05–0.5 Hz, that includes VLP), a broad-band (0.5–10 Hz, that includes explosion-quakes), medium frequencies (1–3 Hz, that includes tremor) and high frequencies (3–10 Hz, that includes landslides).

The analysis for the automatic STA/LTA trigger (Figs. 9–11) was performed with standard routines included in Obspy^44^. We filtered the strainmeter signal, which has a sampling rate of 1 sps, in the 0.0009–0.009 Hz frequency band and we used a Long-Term window of 2700 seconds and a Short-Term window of 900 seconds. We applied a threshold of 2.2 for the value of the STA/LTA ratio.

## Supplementary information

Supplementary information.

## Data Availability

The data time series analyzed in this study are available as Supplementary Data.
