# Supplementary material for: Geophysical precursors of the July-August 2019 paroxysmal eruptive phase and their implications for Stromboli volcano (Italy) monitoring
Source: Sci Rep. 2020 Jun 24;10:10296. doi: 10.1038/s41598-020-67220-1 (PMC7314781; doi:10.1038/s41598-020-67220-1)
Supplement: Supplementary file 1 — Supplementary information. [file 41598_2020_67220_MOESM1_ESM.zip › supplementary_data/supplementary_data.pdf]

Geophysical precursors of the July-August 2019 paroxysmal eruptive phase and their implications for Stromboli volcano (Italy) monitoring

Flora Giudicepietro[1,*], Carmen López[2], Giovanni Macedonio[1], Salvatore Alparone[3], Francesca Bianco[1], Sonia Calvari[3], Walter De Cesare[1], Dario Delle Donne[1], Bellina Di Lieto[1], Antonietta M. Esposito[1], Massimo Orazi[1], Rosario Peluso[1], Eugenio Privitera[3], Pierdomenico Romano[1], Giovanni Scarpato[1], Anna Tramelli[1]

[1]Istituto Nazionale di Geofisica e Vulcanologia, Osservatorio Vesuviano, Napoli, Italy
[2]Observatorio Geofísico Central, Instituto Geográfico Nacional (IGN), Madrid, Spain
[3]Istituto Nazionale di Geofisica e Vulcanologia, Osservatorio Etneo, Catania, Italy
[*]Corresponding author: Flora Giudicepietro, flora.giudicepietro@ingv.it

Supplementary Data

The attached archive `supplemental_data.zip` contains the time series of the different parameters shown in Fig.11 of the paper. Date/time span between November 15, 2018 and September 15, 2019. Data are collected in six ASCII files in comma separated values (csv) format. In each file the first record is the header and the following records contain the data. The header contains comma separated descriptions of the record fields. In the following records, the first field represents the date/time and the second (or eventually also the third) field contains the value of the parameter at the given date/time. The date/time is expressed as Excel date, in order to simplify the use by the common worksheets (MS Excel, OpenOffice, etc.). We remind that the Excel date/time is a real number representing the number of day since December 30, 1899 at 00:00. Missing data are skipped.

The `zip` archive contains the following files inside directory `supplemental_data`:

- File `fractal_dimension.csv` contains the time series of the fractal dimension of the seismic signal recorded at the station STRA (see Fig.1), East component, evaluated in half-hour windows.
- File `mean_sq_ampl.csv` contains the mean square amplitude of the 3-components signal module of the STRA station, evaluated in half-hour windows.
- File `polarization.csv` contains the time series of the polarization of the seismic signal of the station STRA (see Fig.1). Each records has three fields: the date, the azimuth in degrees from North counted clockwise, and the angle of incidence in degrees counted from horizontal and positive upwards. The polarization is evaluated in half-hour windows.
- File `vlp_peak_ampl.csv` contains the maximum VLP peak-to-peak amplitude in half-hour windows. The procedure for evaluating this amplitude is described in the paper.
- File `vlp_per_hour.csv` contains the time series of the daily average of the number of VLPs per hour.
- File `vlp_size.csv` contains the time series of the new parameter "VLP size", evaluated in half-hour windows. The procedure for eavluating the VLP size is described in paper.
